# Clinical value of genetic analysis in prenatal diagnosis of short femur

**DOI:** 10.1002/mgg3.978

**Published:** 2019-09-30

**Authors:** Jialiu Liu, Linhuan Huang, Zhiming He, Shaobin Lin, Ye Wang, Yanmin Luo

**Affiliations:** ^1^ Department of Obstetrics & Gynecology The First Affiliated Hospital of Sun Yat‐Sen University Guangzhou People's Republic of China

**Keywords:** chromosome microarray analysis, fetal femur length, gene sequencing, prenatal diagnosis

## Abstract

**Background:**

Fetal femur length (FL) is an important biometric index in prenatal screening. The etiology of short femur is diverse, with some pathogenic causes leading to adverse outcomes. To improve the accuracy and practicability of diagnosis, we investigated the value of genetic analysis in prenatal diagnosis of short femur.

**Methods:**

We examined chromosomal microarray analysis (CMA) (64 fetuses) and karyotyping (59 fetuses) data retrospectively for short femur without fetal growth restriction (FGR). Genetic testing was conducted for 15 fetuses.

**Results:**

Karyotyping and CMA detected chromosomal aberrations at rates of 13.6% and 27.2%, respectively. Among fetuses with other abnormalities, detection rates were 21.0% higher with CMA than karyotyping. CMA identified chromosomal abnormalities in 36.4% of cases with a FL 2–4 standard deviations (SDs) below the gestational age (GA) mean. Abnormality detection by CMA reached 38.5% in the second trimester. Duplication of 12p, 16p13.1 deletion, and uniparental disomy 16 were identified by CMA in three cases of short femur. Gene sequencing detected clinically notable mutations in 12/15 fetuses, among which 9/12 fetuses had FLs >4 SDs below the GA mean.

**Conclusions:**

CMA yielded a higher detection value than karyotyping in fetuses with other abnormalities or a FL 2–4 SDs below the GA mean during the second trimester. Gene sequencing should be performed when FL is >4 SDs below the mean.

## 1 INTRODUCTION

As an important biometric measurement in prenatal ultrasound (US) screening, fetal femur length (FL) is used to assess gestational age (GA), fetal growth conditions, and skeletal development (Speer, Canavan, Simhan, & Hill, 2014). The length of fetal long bones can be associated with race, ethnicity, and familial tendency (Kasraeian, Shahraki, Asadi, Vafaei, & Sameni, 2017). Pathological short femur may be ascribed to fetal growth restriction (FGR), chromosomal abnormalities, or skeletal dysplasia, all of which can lead to adverse outcomes (Kaijomaa, Ulander, Ryynanen, & Stefanovic, 2016). Additionally, prior studies have suggested placental dysfunction was one of the etiologies of short fetal long bones, accompanied by FGR, preterm birth, or hypertensive pregnancy disorders (Mailath‐Pokorny, Polterauer, Worda, Springer, & Bettelheim, 2015). Although most fetuses with short femur have normal birth outcomes, short FL is nevertheless a soft marker of fetal abnormalities because of the significant risk of adverse pregnancy and neonatal outcomes due to aneuploidy and lethal skeletal dysplasia (Mathiesen, Aksglaede, Skibsted, Petersen, & Tabor, 2014). Therefore, short femur needs to be assessed before birth and diagnosed accurately.

Several molecular‐level invasive prenatal diagnosis approaches are widely applied. Karyotyping reveals chromosomal numerical abnormalities and structural chromosomal aberrations affecting large segments (>5–10 Mb). Chromosomal microarray analysis (CMA) can detect genome‐wide copy number variants (CNVs) and loss of heterozygosity that may account for some abnormal fetal phenotypes (Stosic, Levy, & Wapner, 2018). Some fetuses with short long bones caused by skeletal dysplasia are confirmed to have a monogenic disease that cannot be detected by CMA or karyotyping (Toru et al., 2015). Thus, genetic mutation testing and whole exome sequencing have begun to be used to detect genetic skeletal developmental disorders.

There are limited data in the literature regarding abnormal CMA findings in fetuses with short femur and it has not been established which diagnostic approach can detect the etiology of short femur with the best accuracy. The aim of this study was to explore how to select the most appropriate method for prenatal diagnosis in the context of short femur. We analyzed 64 fetuses defined as having short femur by US retrospectively to evaluate the clinical value of using each genetic analysis method in prenatal diagnosis.

## 2 MATERIALS AND METHODS

### 2.1 Ethical compliance

This study was approved by the Ethics Committee of the First Affiliated Hospital of Sun Yat‐sen University (ID: [2018]227).

### 2.2 Case collection

We studied fetuses with short femur retrospectively at the First Affiliated Hospital of Sun Yat‐Sen University from June 2013 to November 2018. Inclusion criteria required a definite ultrasonic diagnosis of short femur for GA and a complete record of invasive prenatal diagnosis undergoing in our hospital. GA was confirmed by the last menstrual period and US scan at 11–13 + 6 weeks GA. Fetuses with evidence of FGR or selective intrauterine growth restriction (sIUGR) were excluded. A total of 64 fetuses with short femur were enrolled in the study. Among them, the affected fetuses were in singleton pregnancies in 60 cases, and individual fetuses of twin pregnancies in four cases.

**Figure 1:**
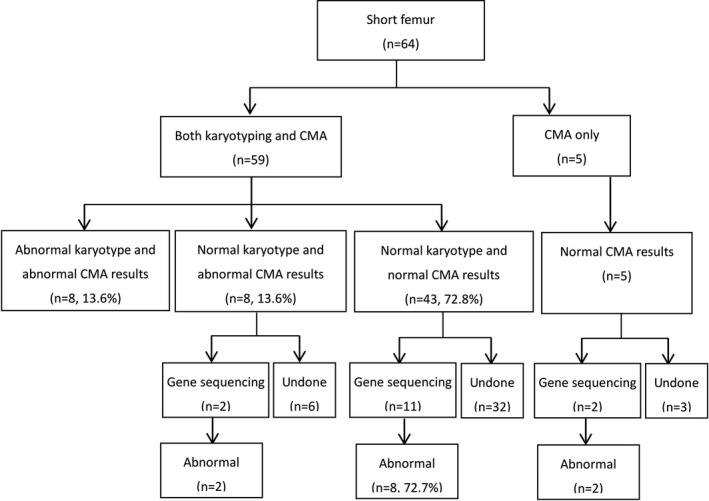
Karyotyping, CMA, and gene sequencing results obtained for fetuses with short femur

All fetuses underwent invasive prenatal diagnostic testing by collecting amniotic fluid via amniocentesis or umbilical cord blood via cordocentesis. Diagnostic testing was by CMA (64 cases), with (59 cases) or without karyotyping. Gene sequencing, which was considered appropriate when fetal skeletal dysplasia was suspected, was completed in 15 fetuses (Figure 1). Pregnancy outcomes were obtained from delivery records (if the mother delivered in our hospital) or from a follow‐up by telephone.

### 2.3 Diagnostic Standards

Based on fetal biometry in the Chinese population (Zhang et al., 2017), short femur was diagnosed if the US measurement of fetal FL was below 2 standard deviations (SDs) of the mean FL according to GA. All US results were obtained in our hospital. To compare the degree of femur shortening, Z‐score was calculated as (X_GA_‐M_GA_)/SD_GA_, where X_GA_ is the US measurement of fetal femur, and M_GA_ and SD_GA_ are the mean value and *SD* at the corresponding GA in weeks.

FGR was defined as the estimated fetal weight (EFW) below the 10th percentile for GA ("ACOG Practice Bulletin No. 2019: Fetal Growth Restriction", 2019), according to the Chinese population references (Cheng, Lu, Leung, Chan, & Sahota, 2018). sIUGR in twin pregnancy was defined as the EFW of one fetus below the 10th percentile and the co‐twin in the normal range ("Management of Monochorionic Twin Pregnancy: Green‐top Guideline No.51", 2017). And EFW was calculated by the formula of Hadlock C based on the US measurement of fetal biometrics (Hadlock, Harrist, Sharman, Deter, & Park, 1985).

### 2.4 Karyotype analysis

Amniotic fluid (30 ml) or umbilical cord blood (1 ml) was obtained by amniocentesis or cordocentesis, respectively, with informed consent. Samples were cultured, passaged, fixed, and prepared for Giemsa‐banding according to a standard cytogenetic protocol. Karyotype analysis and description were based on the International System for Human Cytogenetic Nomenclature (Slovak, Theisen, & Shaffer, 2013).

### 2.5 Chromosomal microarray analysis

Amniotic fluid (10 ml) or umbilical cord blood (1 ml) was collected, and genomic DNA was extracted with a QIAamp DNA Blood mini kit (Qiagen Inc., Germany). Genome‐wide CNVs and heterozygous deletions were detected with the Affymetrix single‐nucleotide polymorphism (SNP) array detection platform and CytoScan HD chip in accordance with the manufacturer's standard operating procedures. ChAS2.0 software was used to analyze chip data. Marked with more than 50 probes, chromosomal DNA affected at >100 kb length was considered a CNV. CNVs were classified according to American College of Medical Genetics (AGMG) guidelines (Kearney, Thorland, Brown, Quintero‐Rivera, & South, 2011). Clinically significant chromosomal aberrations included pathogenic CNVs and variants of unknown significance (VOUSs).

Gene Sequencing DNA was extracted from umbilical cord blood (1 ml). Targeted capture sequencing or whole exome sequencing was used to detect fetal pathogenic mutations responsible for skeletal dysplasia. Sample DNA was fragmented randomly, purified, and enriched to construct DNA libraries. The capture kit included probes designed specifically to capture skeletal development‐associated genes. Whole exome sequencing was performed with an Illumina standard kit. DNA libraries were sequenced on the NextSeq500 sequencer according to the manufacturer's protocols (Illumina). Burrows‐Wheeler Aligner software (version 0.59) was used to make comparisons with the reference sequence of GRCh37.p10. Sequence variants were interpreted based on ACMG guidelines (Richards et al., 2015). Variants were annotated by referring to online databases, including: the Human Gene Mutation Database, the Locus‐Specific Mutation Database (http://www.hgvs.org/dblist/glsdb.htm), the Consensus Coding Sequences Database (https://www.ncbi.nlm.nih.gov/CCDS), the 1000 Genomes Project Dataset (https://www.ncbi.nlm.nih.gov/variation/tools/1000genomes/), and PubMed (http://www.ncbi.nlm.nih.gov/pubmed).

### 2.6 Statistical analysis

The data were analyzed in SPSS 22.0 software (SPSS Inc.).

## 3 RESULTS

### 3.1 Case characteristics

The mothers in the cohort included 41 primiparas and 23 multiparas with a mean maternal age of 30 ± 5 years (range, 19–43 years). The mean GA when short femur was first detected by US was 28.3 ± 3.7 weeks (range, 21^+2^–35^+1^ weeks). The mean GA when invasive prenatal diagnosis was performed was 29.9 ± 3.3 weeks (range, 22^+2^–35^+2^ weeks). The chorionicities of twin pregnancies were confirmed by the ultrasound image in the first trimester. Twin pregnancies in the study included 3 dichorionic diamniotic twins and 1 monochorionic diamniotic twin pair, which short femur detected in one fetus of each twin pairs. None of the cases presented with a pregnancy complication at the time of diagnosis. Outcomes included 26 elected terminations of pregnancy, 1 selective reduction of a twin pregnancy, 24 term births, 7 preterm births, and 1 neonatal death. Five fetuses were lost to follow‐up.

### 3.2 Comparison of karyotyping and CMA results

All fetuses underwent CMA, and 59 fetuses also underwent karyotyping (Figure 1). Karyotype analysis identified chromosomal aberrations in 13.6% of cases (8/59), compared to 25% of fetuses (16/64) for CMA. Considering only the 59 fetuses that underwent both CMA and karyotyping, CMA detected chromosomal aberrations in 27.2% of cases (13/59).

Karyotyping identified 8 fetuses with chromosomal abnormalities, and the CMA results were consistent with these results. We found five fetuses with trisomy 21, 2 fetuses with mosaic monosomy of chromosome X, and 1 fetus with of a marker chromosome. The two abnormal karyotypes with chromosome X mosaicism were 45, X[14]/46, X, i(X)(q10)[5] and 45, X[35]/46, X, +mat[14]/46, X, i(X) (q10)[10]/47, X, i(X)(q10)×2[3]. Termination of pregnancy or selective reduction was chosen in all these cases. Characteristics and outcomes of these fetuses are summarized in Table 2.

### 3.3 CMA detection of abnormalities in fetusess with normal karyotype

CMA detected additional abnormal results in 13.6% (8/59) of fetuses with a normal karyotype (Table 3). Fetus 9 had a pathogenic CNV of a 235‐kb microdeletion in chromosome 16, which is related to alpha thalassemia intellectual disability syndrome (ATR‐16 syndrome). In fetus 10, microarray confirmed loss of heterozygosity (LOH) in chromosome 16 with a 9.002‐Mb homozygous segment. CMA identified 6 fetuses with VOUSs, associated with microduplications ranging from 106 kb to 6.628 Mb. Relevant OMIM genes included leucine zipper‐like transcriptional regulator 1 (*600574, *LZTR1*), CRK like proto‐oncogene, adaptor protein (*602007, *CRKL*), short stature homeobox (*312865, *SHOX*), nephrocystin 1 (*607100, *NPHP1*), gephyrin (*603930, *GPHN*), retinol dehydrogenase 12 (*608830, *RDH12*), and zinc finger FYVE‐type containing 26 (*612012, *ZFYVE26*), among others.

### 3.4 Karyotype and CMA findings in relation to case characteristics

Among the 59 fetuses that underwent karyotyping and CMA, 38 fetuses (64.4%) had other US abnormalities in addition to short femur, and 21 fetuses (35.6%) had isolated short femur. Karyotyping and CMA both detected chromosomal aberrations in 9.5% (2/21) of the fetuses of isolated short femur. When US was used to screen out additional abnormalities, CMA identified a larger percentage of chromosomal aberrations (36.8%, 14/38) than did karyotyping (15.8%, 6/38). Eleven cases with abnormal soft markers, such as absent nasal bone, thickened nuchal fold, and enlarged lateral ventricles, were detected, with an abnormality detection rate of 27.3% (3/11) for karyotyping and 45.5% (5/11) for CMA. Another 18 fetuses were complicated with structural malformations, mainly involving the cardiovascular and skeletal systems. CMA identified chromosomal abnormalities in a greater number of these 18 fetuses (33.3%, 6/18) than were detected by karyotyping (11.1%, 2/18). Nine fetuses showed abnormal US findings of polyhydramnios, seroperitoneum, or hydropericardium, and 3 fetuses (3/9, 33.3%) showed significant CNVs. Characteristics of these findings are listed in Table 1.

**Table 1:** Relation of karyotype and chromosomal microarray analysis (CMA) findings to case characteristics

Characteristic	*N*	Abnormal karyotype, *n* (%)	Pathogenic CNVs and VOUSs, *n* (%)
US findings			
Isolated short long bones	21	2 (9.5%)	2 (9.5%)
Other US abnormalities	38	6 (15.8%)	14 (36.8%)
Abnormal soft markers	11	3 (27.3%)	5 (45.5%)
Structural malformation	18	2 (11.1%)	6 (33.3%)
Other abnormalities^a^	9	1 (11.1%)	3 (33.3%)
Z‐score			
−4 ≤ *Z*‐score ≤−2	44	8 (18.2%)	16 (36.4%)
*Z*‐score <−4	15	0 (0.0%)	0 (0.0%)
GA at initial diagnosis			
All second trimester	26	5 (19.2%)	10 (38.5%)
Second trimester, ≤24 weeks	12	4 (33.3%)	6 (50.0%)
Second trimester, 24–28 weeks	14	1 (7.1%)	4 (28.6%)
All third trimester	33	3 (9.1%)	6 (18.2%)

Abbreviations: CNVs, copy number variations; GA, gestational age; US, ultrasound; VOUSs, variants of unknown significance.

a Other abnormalities include polyhydramnios, seroperitoneum, and hydropericardium.

The Z‐score was calculated to reflect the degree of femur shortening, using diagnostic standards and the exact GA in weeks. Of the 59 fetuses that underwent both karyotyping and CMA, 44 (74.6%) fetuses had Z‐scores between −2 and −4, and 15 (25.4%) fetuses had Z‐scores below −4. All abnormal karyotyping and CMA results occurred in fetuses with FL between −2 and −4 *SD* below the GA mean, with detection rates of abnormal results of 36.4% (16/44) by CMA and 18.2% (8/44) by karyotyping.

Short femur was first diagnosed in the second trimester for 26 fetuses and in the third trimester for 33 fetuses. Karyotyping detected chromosomal aberrations in the second trimester in 19.2% of fetuses (5/26), whereas the CMA detection rate in this period was 38.5% (10/26). When initial diagnosis was performed before 24 weeks GA, the detection rate of chromosomal abnormalities increased to 50.0%. In the third trimester, CMA had a higher detection rate of chromosomal aberrations (6/33, 18.2%) than did karyotyping (3/33, 9.1%).

### 3.5 Pathogenic results detected by gene sequencing

Fifteen fetuses successfully underwent gene sequencing of skeletal development or whole exome sequencing. We found pathogenic aberrations in 12 fetuses (80.0%) (Table 4). Only CMA detected a segmental LOH in chromosome 16 and a VOUS with an 865‐kb microduplication on 2q13. Positive gene sequencing results revealed significant mutations on six genes: fibroblast growth factor receptor 3 (*134934, *FGFR3*), collagen type I alpha 2 (*120160, *COL1A2*), collagen type II alpha 1 (*120140, *COL2A1*), pericentrin (*605925, *PCNT*), biotinidase (*609019, *BTD*), and solute carrier family 25 member 13 (*603859, *SLC25A13*). Among the potentially pathogenic aberrations affecting 12 fetuses, 7 were known to be pathogenic, 2 were likely pathogenic, and 3 were of unknown significance. These genetic aberrations may be associated with osteopsathyrosis, achondroplasia, lethal skeletal dysplasia, or other diseases. Of the 12 fetuses with significant genetic mutations, 8 (66.7%) had additional malformations detectable on US, including skeletal morphological abnormalities or dysmorphic facial features in 6 fetuses (50.0%). In all 9 fetuses with a Z‐score less than −4, pathogenic or likely pathogenic genetic mutations were observed.

## 4 DISCUSSION

Although previous studies have demonstrated clear advantages of CMA in prenatal diagnosis (Committee on Genetics & the Society for Maternal‐Fetal Medicine, 2016), very little research has addressed short long bone associations with CMA findings. Here, we found that CMA had a higher detection rate of chromosomal abnormalities than karyotyping (25.0% vs. 13.6%) in fetuses with short femur. Our detection rate with karyotyping was similar to the 16% rate reported by Beke et al., (2005). Our CMA detection rate was 21.0% higher than that of karyotyping among cases with additional US abnormalities. CMA identified more aberrations in fetuses with non‐isolated short femur. Short femur was much more likely to be related to chromosomal aberrations when other soft markers were observed than when isolated short femur was seen. These findings highlight the added value of CMA in fetuses of short femur, especially when additional US abnormalities are observed.

Short femur is a sonographic soft marker of aneuploidy, especially for Down syndrome (DS) (Benacerraf, Neuberg, Bromley, & Frigoletto, 1992; Bethune, 2007). Our study supports the contribution of short femur for DS (Agathokleous, Chaveeva, Poon, Kosinski, & Nicolaides, 2013), as we detected 5 DS fetuses (Table 2, No. 1–5), with a high detection rate of 8.5% among all short femur cases. In fetuses 7 and 8, we detected mosaics of monosomy chromosome X by karyotyping, and large fragment deletions and rearrangements in chromosome X by CMA. The clinical manifestations of mosaics were similar to those of Turner syndrome, which is characterized by short statue, premature ovarian failure, and reproductive system dysplasia. Short femur is a typical finding of Turner syndrome in prenatal US screening (Papp et al., 2006).

**Table 2:** Abnormal karyotype and abnormal chromosomal microarray analysis (CMA) result in fetuses with short femur

No.	*Z*	Other US malformations	Karyotype	CMA results	Size (Mb)	Diag.	Outcome
1	−2.1	Atrial septal defect, ventricular septal defect, bilateral enlarged lateral ventricles	47, XX, +21	21q11.2q22.3 (15016486–48093361)×3	33.07	T21	TOP 22^+3^ w; wideset eyes, protruding tongue, BL 48 cm, BW 2.28 kg
2	−2.4	Absent nasal bone, echogenic bowel, high hepatic and renal parenchyma, complete endocardial cushion defect, tetralogy of Fallot, bilateral enlarged lateral ventricles	47, XX, +21	21q11.2q22.3 (15190686–48097372)×3	32.91	T21	TOP 30 w
3	−2.3	Hypoplastic nasal bone	47, XY, +21	21q11.2q22.3 (15190686–48097372)×3	32.91	T21	TOP 27^+3^ w
4	−2.2	‐	47, XY, +21	21 × 3		T21	TOP 34 w
5	−2.6	Absent nasal bone, thickened nuchal fold	47, XX, +21	21 × 3		T21	TOP 27^+5^ w
6	−2.8	Polyhydramnios	47, XN, +mar[83]/ 46, XN[17]	12p13.33p11.1 (173786–34835837)×3	34.66	Tetrasomy 12p	TOP 30^+4^ w
7	−3.4	‐	45, X[35]/ 46, X, +mat[14]/ 46, X, i(X)(q10)[10]/ 47, X, i(X)(q10)*2[3]	Xp22.33p11.22 (168546–50874418)×1 Xp11.22q21.1 (50874419–82034738)×1.5–2 Xq21.1q28 (82034739–155233)×1.2	50.71 31.16 73.2	Mosaic	TOP 31^+2^ w
8	−2.4	DCDA, Critically enlarged posterior fossa	45, X[14]/ 46, X, i(X)(q10)[5]	Xp22.33p11.1 (169921–58227320)×1 Xp11.1q28 (58227320–155270560)×1–2	58 97	Mosaic	Selective reduction

Note

As all CMA analysis were run in array form (arr) with Human Genome build 19 (hg19), the notation “arr[hg19]” has been removed from the CMA results.

Abbreviations: BL, body length; BW, body weight; CMA, chromosomal microarray analysis; DCDA, dichorionic diamniotic; T21, trisomy 21; TOP, termination of pregnancy; US, ultrasound; w, weeks; *Z*, *Z*‐score.

In fetus 6, karyotyping indicated a mosaic of the marker chromosome with a ratio of 83%, and CMA detected a 34.66‐Mb duplication on 12p. The 12p‐duplication may present clinical symptoms similar to Pallister‐Killian syndrome, including polyhydramnios and short limbs (Izumi et al., 2012). Through CMA, we defined the origin of the marker chromosome (Levy & Wapner, 2018) and highlighted the effect of CMA on detecting visible cytogenetic abnormalities of unknown origin in karyotype, which can better confirm the diagnosis and facilitate genetic counseling.

Regarding the normal karyotype fetuses in this study, several significant aberrations identified by CMA may account for short femur. In fetus 9, we detected a pathogenic microdeletion of 235 kb on 16p13.1, involving *HBA1* and *HBA2*. This microdeletion is associated with ATR‐16 syndrome, with phenotypes of anemia associated with α‐thalassemia, intellectual disability, skeletal abnormalities, facial dysmorphism, and short stature (Gibbons, 2012). In this case, the pregnancy was terminated at 35^+2^ gestational weeks and the fetus had six fingers on her right hand. In the case of fetus 10, CMA revealed a 9.002‐Mb LOH in chromosome 16. The aberration was further verified by sequencing and SNP analysis with parental samples, which indicated that the abnormal chromosomal variation was derived from maternal uniparental disomy (mUPD) 16, containing a 78.32‐Mb heterodisomy segmental mUPD and an 8.83‐Mb isodisomy segmental mUPD. The clinical implications of mUPD 16 are unclear, but some researchers have reported possible associated phenotypes including FGR, short stature, and feeding difficulties (Abu‐Amero, Ali, Abu‐Amero, Stanier, & Moore, 1999; Yingjun et al., 2017). As the pregnancy progressed, the fetus developed severe FGR and the parents chose to terminate the pregnancy at 34 gestational weeks, at which time the fetus was 1.51 kg. It is our view that that the mUPD16 was the likely cause of FGR, and that short femur was likely an early sign of the FGR.

VOUSs were detected in 6 fetuses (Table 3, No. 11–16). In fetus 11, a 175‐kb microduplication was observed on 22q11.2, a locus that overlaps with variants in DGV database, indicating some individuals in the normal population have this variant. Simultaneously, a search of the ClinGen database revealed this microduplication region at 22q11.2 overlaps partially with a recurrent region that includes the critical gene *CRKL*. Due to there being little evidence for triplosensitivity pathogenicity near this region, its correlation with short femur is uncertain. In fetus 13, our results showed a 106‐kb microduplication on Xp22.33, which includes the single‐dose‐sensitive short stature homeobox gene (*SHOX*.*)*. Mutations and deletions in *SHOX* can lead to Leri‐Weill dyschondrosteosis and idiopathic short statue (Kang, 2017; Oliveira & Alves, 2011). Although clinical significance of this microduplication has not been clarified, three cases of short statue with *SHOX* duplication have been reported (Benito‐Sanz et al., 2011). Thus, *SHOX* microduplication could be responsible for the observed short femur in fetus 13. In fetuses 14 and 15, an 865‐kb microduplication was found at 2q13, which affects *NPHP1*. Mutation or homozygous deletion in *NPHP1* has been reported in patients with genetic syndromes with renal failure, intellectual disability, growth retardation, autism, and language delay (Wolf & Hildebrandt, 2011). However, although some research (Riley et al., 2015) showed behavioral, psychiatric, and developmental delay phenotypes in cases of 2q13 duplication, few reports have confirmed the relationship between *NPHP1* duplication and prenatal short femur. Fetus 12 harbored a 6.628‐Mb duplication at 11q24.3q25, a region that includes 22 OMIM genes. Additionally, Fetus 16 showed a 1.87Mb microduplication on 14q23.3q24.1, involving *GPHN, RDH12,* and *ZFYVE26*. No association between these genes and short long bones has been found. More case studies and additional research on the molecular mechanisms of genes will help to expand our understanding of genotypes and phenotypes associated with fetal short femur.

**Table 3:** Abnormal CMA results of short femur fetuses with normal karyotype

No.	*Z*	Other US malformation	Karyo‐type	CMA results	Size (Mb)	Type	OMIM gene or related disorder	Clinical signif.	Outcome
9	−3.0	Enlarged left lateral ventricle, small lower jaw, ventricular septal defect, thickened nasal cartilage	46, XX	dup4q35.2 (189905481–19095747) ×3 del16p13.1 (85880–320729) ×1	1.05 0.235	Gain Loss	‐ ATR−16 syndrome; *HBA2* (*141,850), *HBA1* (*141,800)	Benign pathogenic	TOP 35^+3^ w, 6 digits on right hand, BL 48 cm, BW 2.28 kg
10	−3.0	Left superior vena cava	46, XX	16q23.2q24.3 (81161763–90163275)×2 hmz	9.002	LOH		Likely pathogenic	TOP, 33w, BL 39cm, BW 1.51 kg
11	−3.2	Bent long bone	46, XX	dup 22q11.2 (21290949–21465659)×3	0.175	Gain	*6* OMIM *genes:* *LZTR1* (*600,574), *CRKL* (*602,007), etc.	VOUS	Term birth 40^+1^w, BL 50cm, BW 3.2 kg
12	−2.1	Hyperechoic focus in the left ventricular	46, XY	dup11q24.3q25 (128231339–134859729)×3	6.628	Gain	*22* OMIM genes	VOUS	Term birth 39w, BL 51 cm, BW 3.3 kg
13	−3.7	Left isolated lung	46, XY	dup Xp22.33 (532444–640818) ×4 dup 1q21.2 (147929322–149660970) ×3 dup 16p11.2 (32554241–33779681) ×3	0.106 1.732 1.225	Gain Gain Gain	*SHOX* (*312,865)	VOUS Benign Benign	TOP 32^+^ w
14	−3.6	Polyhydramnios	46, XY	dup2q13 (110504318–111369233) ×3	0.865	Gain	*NPHP1* (*607,100)	VOUS	TOP 29^+4^ w
15	−2.8	MCDA, short long bones, echogenic bowel, polyhydramnios, seroperitoneum	46, XY, 22pss	dup2q13 (110504318–111369264) ×3	0.865	Gain	*NPHP1* (*607,100)	VOUS	Preterm birth 36^+5^ w, BW 2.3 kg, died 10 d after birth
16	−2.3	Arachnoid cyst, tricuspid regurgitation	46, XY	dup 14q23.3q24.1 (67385727– 69,259,088) ×3	1.87	Gain	*GPHN* (*603,930), *RDH12* (*608,830), *ZFYVE26* (*612,012)	VOUS	Preterm birth 34^+5^w, BL 53 cm, BW 3.2 kg

Note

As all CMA analyses were run in array form (arr) with Human Genome build 19 (hg19), the notation “arr[hg19]” has been removed from the CMA results.

Abbreviations: BL, body length; BW, body weight; CMA, chromosomal microarray analysis; hmz, homozygous; LOH, loss of heterozygosity; MCDA, monochorionic diamniotic; TOP, termination of pregnancy; US, ultrasound; VOUS, variants of unknown significance; w, weeks; Z, Z‐score.

Comparing the GA of initial diagnosis among fetuses with short femur, we hypothesized that a greater number of chromosomal abnormalities would be identified during the second than during the third trimester. The chance of chromosomal abnormalities was higher when short femur was detected earlier, with a 50.0% chromosomal abnormality detection rate by CMA when assessed before 24 gestational weeks.

**Table 4:** Characteristics of fetuses with short femur and pathogenic gene mutation

No.	*Z*	Other US malformations	Karyo‐ type	CMA results	Gene sequencing results			Related disorder(s)	Outcome
					Genes	NT, predicted AA changes	Mutation type, inheritance		
10	−3.0	Left superior vena cava	46, XX	16q23.2q24.3′2 hmz, 9.002 Mb	16p13.3q23.1 16q23.2q24.3 *PCNT PCNT*	78.32Mb 8.83Mb c.1040A>G, P.K347R c.8960G>A, p.R2987Q	mUPD, Het, likely pathogenic mUPD, Isodisomy, likely pathogenic Het, inherited from father, unknown significance, AR Het, inherited from mother, unknown significance, AR	mUPD16 Microcephalic osteodysplastic primordial dwarfism, type II	TOP 34w, BL 39 cm, BW 1.51 kg
14	−3.6	Polyhydramnios	46, XY	Dup 2q13 VOUS	*BTD SLC25A13*	c.420 G>A, p.W140 Ter c.852‐855 delTATG, p.R284RfsX3	Both Het, unknown source, likely pathogenic, AR	Biotinidase deficiency; citrullinemia	TOP 29^+4^ w
17	−4.1	Thickened nuchal fold, unclear nasal bone	46, XX	Normal	*FGFR3*	c.1138G>A, p.G380R	Het, de novo, pathogenic, AD	Achondroplasia	Term birth, BL 50 cm, BW 3.7 kg
18	−6.2	Protruding forehead, collapsed nasal root	46, XY	Normal	*FGFR3*	c.1138 G>A, p.G380R	Het, de novo, pathogenic, AD	Achondroplasia	TOP 32^+5^ w
19	−8.0	‐	‐	Normal	*FGFR3*	c.1138 G>A, p.G380R	Het, unknown source, pathogenic, AD	Achondroplasia	TOP 34 w, BL 35 cm, BW 1.79 kg
20	−10.6	Protruding forehead, abnormal head, short limbs, bent and narrow chest	46, XY	Normal	*FGFR3*	c.1118 A>G, p.Y373C	Het, de novo, pathogenic, AD	Lethal skeletal dysplasia type I	TOP 22^+6^ w, BL 27 cm, BW 0.64 kg, short limbs, abdominal bulging, narrow chest, bell‐shaped thorax.
21	−7.2	Narrow chest	46, XX	Normal	*FGFR3*	c.1144G>A, p.G382R	Het, de novo, pathogenic, AD	Achondroplasia	TOP 33w
22	−4.1	‐	46, XY	Normal	*COL2A1*	c.1636G>A, p.G546S	Het, de novo, pathogenic, AD	Developmental hip dysplasia, spinal osteochon‐ drosis, etc.	TOP 33w
23	−5.1	‐	‐	Normal	*COL2A1*	c.1070 G>C, p.G357A	Het, de novo, likely pathogenic, AD	Achondroplasia type II, Czech dysplasia, etc.	TOP 27 w
24	−5.3	Narrow chest, short and bent limbs, short ribs, Blake's pouch cyst, polyhydramnios	46, XY	Normal	*COL2A1*	c.4A>T, p.I2F	Het, unknown source, unknown significance, AD	Short‐rib polydactyly syndrome	TOP 25^+1^w, BL 35 cm, BW 0.89 kg, 6 digits on both hands and foots
25	−3.5	Polyhydramnios, cardiomegaly, hydropericardium	46, XX	Del 16p11.2 Benign	*COL1A2*	c.2330 G>A, p.R777H	Het, de novo, unknown significance, AD	Osteopsathy‐ rosis	TOP 34^+2^ w, BL 41 cm, BW 2.21 kg
26	−4.9	Bent long bone, uneven ribs	46, XY	Normal	*COL1A2*	c.2565+1 G>A, p.?	Het, de novo, pathogenic, AD	Osteopsathy‐ rosis	TOP 28 w, BL 33 cm, BW 0.99 kg

Abbreviations: AA, amino acid; AD, autosomal dominant; AR, autosomal recessive; BL, body length; BW, body weight; CMA, chromosomal microarray analysis; Del, deletion; Dup, duplication; fs, frame shift; het, heterozygous mutations; NT, nucleotide; TOP, termination of pregnancy; Ter, terminator codon mutation; US, ultrasound; VOUS, variants of unknown significance; w, weeks; Z, Z‐score.

Gene sequencing revealed clinically important mutations not found by karyotyping or CMA in 12 fetuses. In fetuses 17–21, the following respective pathogenic mutations affecting *FGFR3* were identified: c.1138G>A (*N* = 3), c.1118A>G (*N* = 1), and c.1144G>A (*N* = 1). Previous studies have confirmed the pathogenicity of these variants in skeletal dysplasia, and c.1138G>A is the most common *FGFR3* mutation underlying achondroplasia (Chen et al., 2017); (Foldynova‐Trantirkova, Wilcox, & Krejci, 2012). Three fetuses were found to have heterozygous mutations of *COL2A1* (c.1636G>A, c.1070 G>C, c.4A>T), which have been associated with various types of osteogenic dysplasia (Deng, Huang, & Yuan, 2016). (Al Kaissi et al., 2013) described a patient with a c.1636G>A mutation of *COL2A1* who presented with hip dysplasia and spinal osteochondritis. The *COL2A1* mutation c.1070G>C, which was found in fetus 23, has been associated with protein effects by bioinformatics predictive analysis, leading us to conclude that this mutation is likely pathogenic, although further study is needed. US screening revealed that fetus 24 had a narrow chest, short ribs, and short bent limbs. Sequencing analysis of fetus 24’s genome demonstrated the presence of the c.4A>T *COL2A1* mutation, but the clinical significance of this mutation has yet to be described in the literature. Following termination of pregnancy, it was discovered that the fetus had six digits on both hands and both feet. Ultimately, we diagnosed fetus with short‐rib polydactyly syndrome and inferred that this mutation might be the reason for the abnormal skeletal development. *COL1A2*, located at 7q21.3, encodes the alpha‐2 (I) chain of type I collagen. Mutation of *COL1A2* (fetuses 25 and 26) is thought to be associated with osteogenesis imperfecta, a congenital disease characterized by fragile bones, multiple fractures, blue sclera, dentinogenesis imperfecta, and deafness (Marini et al., 2017; Zhang et al., 2016).

In addition to mUPD 16, fetus 10 had two heterozygous missense mutations in *PCNT*, having inherited one from each parent. Compound heterozygous mutations in *PCNT* cause microcephalic osteodysplastic primordial dwarfism type II, which is characterized by intrauterine growth retardation, severe proportionate short stature, and microcephaly (Rauch et al., 2008). Neither of the heterozygous *PCNT* mutations found in fetus 10 has been reported in clinical cases previously. However, it is noteworthy that one of these mutations, p.R2987Q, is at a low‐conservation position. Given the insufficiency of evidence that would suggest that short femur pathogenicity could be attributed to these two missense mutations, it is more probable that the phenotype was related, at least in part, to the mUPD 16. In fetus 14, we detected likely pathogenic mutations in *BTD* and *SLC25A13*, but both heterozygous mutations were inherited in an autosomal recessive pattern. Therefore, we considered them to be negative results for short femur.

Skeletal dysplasia disorders are usually accompanied by multiple US abnormalities, especially extremely short femur, abnormal skeletal shape, and other atypical facial characteristics (Milks, Hill, & Hosseinzadeh, 2017; Victoria et al., 2018). Most are monogenic conditions but cannot be detected by karyotyping or CMA. Therefore, when a prenatal US scan shows that the fetal femur is extremely short, it should be determined whether there are other skeletal morphological abnormalities or unique characteristics.

Importantly, we found that all chromosomal aberrations were detected in fetuses with a Z‐score between −2 and −4. Consistent with previous literature (Huang et al., 2007; Ren et al., 2017), we concluded that fetuses with FLs two to four SDs below the mean FL for GA may be more likely to have chromosomal aberrations. Of the 15 fetuses that underwent gene sequencing, 9 had Z‐scores below −4, and all had pathogenic mutations; of the remaining 6 fetuses with Z‐scores between −2 and −4, only 1 had a pathogenic mutation accounting for short femur. For fetuses with extremely short femur (FL>4 *SD* below GA mean), the possibility of skeletal dysplasia should be considered (Victoria et al., 2018).

Various US findings, different degrees of femur shortness, and clinical characteristics of short femur may be attributed to different genetic abnormalities. Because microarray analysis and gene sequencing can be cost prohibitive, the most appropriate gene sequencing method given the clinical signs observed should be pursued. For fetuses with FLs between 2 and 4 *SD* below GA mean, karyotyping, and CMA are recommended for detecting chromosomal malformations. To improve the accuracy of prenatal diagnosis, we strongly recommend that gene sequencing be used for prenatal diagnosis in fetuses with extremely short femur or skeletal morphological abnormalities.

Our study had some limitations. As a retrospective analysis, our clinical sample was relatively small. Prospective multicenter studies with large sample sizes are needed to obtain more reliable data. Secondly, some cases that underwent prenatal diagnosis in our hospital were delivered or induced in other local hospitals, resulting in incomplete follow‐up information.

In conclusion, compared to karyotyping, CMA had higher detection rate of clinically significant CNVs in fetuses with additional US abnormalities and in fetuses with FLs between 2 and 4 SDs below GA mean during the second trimester. This work underscores the importance of performing gene sequencing on fetuses with extremely short femur (FL>4 SDs below GA mean). Our research may aid in early prenatal diagnosis and genetic counseling for fetal short femur.

## CONFLICT OF INTEREST

The authors declare no conflicts of interest.

## STATEMENT OF ETHICS

This study was approved by the Ethics Committee of the First Affiliated Hospital of Sun Yat‐sen University.
